# Knowledge and Attitude of Faculty Members Working in Dental Institutions towards the Dental Treatment of Patients with HIV/AIDS

**DOI:** 10.1155/2014/429692

**Published:** 2014-10-28

**Authors:** Sukhvinder Singh Oberoi, Nilima Sharma, Vikrant Mohanty, Charumohan Marya, Amit Rekhi, Avneet Oberoi

**Affiliations:** ^1^Public Health Dentistry Department, Sudha Rustagi College of Dental Sciences and Research, Faridabad, Haryana, India; ^2^Pandit Bhagwat Dayal Sharma University, Rohtak, Haryana, India; ^3^Department of Dentistry, Hamdard Institute of Medical Sciences and Research, New Delhi, India; ^4^Hamdard University, New Delhi, India; ^5^Public Health Dentistry Department, Maulana Azad Institute of Dental Sciences, BS Zafar Marg, New Delhi, India; ^6^Public Health Dentistry Department, Sudha Rustagi Dental College, Faridabad, India; ^7^Pandit Bhagwat Dayal Sharma University, Rohtak, India; ^8^Oberoi Dental Clinic and Orthodontic Centre, Tagore Garden, New Delhi, India

## Abstract

*Background*. Dentists have an ethical responsibility to provide treatment to HIV-infected patients, particularly because oral lesions are common among these patients. However, there are no official guidelines as to how to treat people living with human immunodeficiency virus (HIV)/acquired immunodeficiency syndrome (AIDS) (PLWHA) or how to screen for potentially infectious people. *Materials and Method*. A descriptive cross-sectional questionnaire based study which assessed the knowledge and attitude of the faculty members towards the treatment of patients with HIV/AIDS was carried out in the Sudha Rustagi College of Dental Sciences, Faridabad, and Maulana Azad Institute of Dental Sciences, New Delhi. *Results*. The willingness to treat patients with HIV was found to be 86.0% among the faculty members in the present study. The majority (79%) of the faculty members thought that treating an HIV-positive patient is ethical responsibility of the dentist. There was a positive attitude (88.0%) among faculty members that routine dental care should be a part of the treatment of patients with HIV/AIDS. *Conclusion*. The level of knowledge regarding HIV and AIDS was acceptable in the present study. However, continuing dental education (CDE) programmes should be conducted on a regular basis for updating the knowledge level of the faculty members towards the dental treatment of patients with HIV/AIDS patients.

## 1. Introduction

The AIDS epidemic is continuing to grow; global estimates have indicated that over 40 million people are infected [1]. The important issue is that the number of newly reported HIV infection cases is increasing every year with a changing epidemic pattern but dental care workers are unprepared to treat this increasing number of people living with HIV/AIDS (PLWHA) [2].

Oral health is an essential aspect of overall medical care for individuals with HIV [3]. Oral care for HIV-positive individuals plays a vital role in improving their nutritional intake, medication tolerance/effectiveness, treatment success rate, and quality of life [4]. With improved survival rates, it is expected that more HIV-positive patients, with or without knowledge of their serologic status, will be seeking dental care in the near future [5].

It is essential that every effort be made to protect both health care workers and patients from HIV exposure in the dental practice as the usual route of transmission is through an individual's contact with infected blood or other bodily fluids [6]. The previous reports have indicated that about 90% of the HIV infections among healthcare workers occur in developing countries where occupational safety is a neglected issue [7–9].

In one of the studies by Samaranayake et al. in UK and USA, a significant proportion of respondents in both countries said they will not attend their dentist if the latter treats AIDS patients and significantly more Americans thought that AIDS transmission was likely in the dental clinic. An overwhelming majority thought that specially trained dentists should be employed to treat AIDS patients while the majority of Americans surmised that AIDS is a serious threat to public health [10]. A negative trend is seen towards the AIDS patients as well as the dentists treating the AIDS patients.

Dental faculty can play an important role as a role model for the dental students (as dentists of the future) regarding the dental treatment of HIV/AIDS patients as students generally follow the teaching of their teachers [11, 12]. Several studies have been conducted to assess the willingness of the oral health professionals to treat HIV-positive individuals either as a main survey or as a part of the knowledge, attitudes, and practice survey in many parts of the world such as Brazil (1994) [13], Mexico (1998) [14], Jordan (2005) [15] and Kuwait and Srilanka (2011) [16]. All these studies have demonstrated a satisfactory knowledge level among the study population considered but a stigma regarding the treatment of HIV/AIDS patients has been reported in the majority of the studies.

The studies for assessing the attitude and knowledge of the dental students regrading the dental treatment of HIV/AIDS patients have been conducted in India by Aggarwal and Panat in Bareilly, Uttar Pradesh in 2013 [17] and Fotedar et al. in Shimla in 2012 [18]. These studies have shown good knowledge score among the dental students (68.3 percent in the study by Fotedar et al. [18] and 78.8 percent by Aggarwal and Panat [17]). As such, no gender related differences have not been reported in the literature in the studies by Aggarwal and Panat [17] and Fotedar et al. [18].

But no published study has been reported in the literature assessing the knowledge and attitude of the faculty members working in teaching institutions where they have a responsibility of both teaching the students and treating the patients. Thus, the present study was carried out to assess the knowledge and attitude of faculty members working in teaching institutes towards the dental treatment of HIV/AIDS infected individuals and also to explore the gender differences in the knowledge and attitude among the faculty members that may exist.

## 2. Materials and Method

The present study was a descriptive cross-sectional questionnaire based study which assessed the knowledge and attitude of faculty members regarding the dental treatment of HIV/AIDS patients. The study was carried out at Maulana Azad Institute of Dental Sciences, New Delhi, India, and Sudha Rustagi College of Dental Sciences, Faridabad.

All the faculty members in both the teaching institutions were full time faculty members as per the Dental council of India (DCI) regulations. AS per DCI regulations, these faculty members are involved in classroom teaching, pre-clinical training and clinical teaching and have designated schedule to be involved in these activities.

### 2.1. Ethical Clearance

The permission to carry out the study was obtained from the director-principal of the institute. The ethical committee clearance was taken from the institutional ethical committee. The study was explained to the faculty members working in these institutes and informed consent was taken from the respondents who agreed to participate in the study.

### 2.2. Questionnaire

The Questionnaire used for the study was a structured Questionnaire which contained questions pertaining to the knowledge and attitude of the faculty members. The questionnaire was formulated after the review of other questionnaires used in the literature by Aggarwal and Panat [17], Fotedar et al. [18] and Manz et al. [19] for the assessment of knowledge and attitude among the dental practitioners and dental students. The attitude related questions assessed the willingness of the faculty members towards the dental treatment of HIV/AIDS patients, the potential barriers they perceived and infection control procedures in the treatment of the HIV/AIDS patients. A 3-point Likert scale was used for assessing the attitude of the respondents pertaining to these questions.

The knowledge level of the faculty members regarding the infection control procedures and oral manifestations was assessed in the form of responses as Yes/No/Don't Know. The response to immediate measures to be taken after a needlestick injury and risk of contracting HIV infection from an HIV-contaminated needlestick injury was also assessed.

### 2.3. Pilot Study

The structured questionnaire used in this study was pilot tested on 20 faculty members from the 2 dental institutions who were not included as a part of the final study sample. Use of words and clear understanding of the questions were checked. After pilot testing, the questionnaire wording was amended wherever required.

### 2.4. Data Collection

The survey questionnaire was distributed to the faculty members by a single examiner and explained thoroughly. A reminder was given to the faculty members after 2 days and the survey questionnaire was collected after 7 days. The questionnaire was administered to 127 faculty members, of which, 100 subjects participated in the study with a response rate of 78.74%. The response rate from the 2 institutions was 75.41% and 81.81% respectively.

### 2.5. Statistical Analysis

The data were tabulated and entered into Microsoft excel 2010 and analysed using the SPSS software 16.0. The descriptive analysis of the responses was carried out and the results were expressed in the form of frequencies and percentages. The chi-square test was used for comparison between males and females with respect to the questions related to knowledge and attitude regarding the treatment of HIV/AIDS patients.

## 3. Results

The mean age of the study population was 36.99 ± 4.95 and consisted of 56 (56%) male and 44 (44%) female faculty members. The mean practice years of the study population was 8.41 ± 4.76. There were 46 (46%) respondents from the Maulana azad institute of dental sciences and 54 (54%) from Sudha Rustagi College of Dental Sciences, Faridabad. The majority of the respondents were involved in both institutional and private practice (54%) whereas only (46%) respondents were involved in the institutional practice only. No significant difference was found among the respondents only in institutional practice and respondents with both institutional and private practice.

The responses from the two institutions were compared and no significant difference was found with respect to the various questions related to the knowledge and attitude among the faculty members from the 2 institutions.

The willingness to treat patients with HIV/AIDS was high (86.0%) among the faculty members of the dental colleges in the present study. Majority (79.0%) of the faculty members agreed that treating an HIV-positive patient is an ethical responsibility of the dentist, whereas 12.0% respondents did not agree. A comparatively higher number of males (85.5%) in comparison to females (71.1%) agreed that treating an HIV-positive patient is an ethical responsibility of the dentist (Table 1).

Thirty-two percent of the dentists had an opinion that treating an HIV-positive patient places a dentist at an increased risk of HIV infection while 66% respondents disagreed. There was a positive attitude among the faculty members towards the dental treatment of HIV/AIDS. Majority (72.0%) of the dentists agreed that they will take dental treatment if required from a dentist who is treating the HIV-positive patients whereas 21% respondents disagreed. A large majority (64.0%) of the faculty members agreed that dealing with the staff/assistant fear towards patients with HIV/AIDS would be a potential barrier in treating patients with HIV/AIDS (Table 1).

Most of the faculty members (70.0%) did not agree that infection control procedures are time consuming and may affect the work quality of the practitioner. Majority (88%) of the faculty members were in favour of the fact that dental treatment should be a part of the treatment of patients with HIV/AIDS. Majority (89.0%) of the dentists agreed that they carried out the treatment of their routine patients as if they were potentially infectious for HBV or HIV infection (Table 1).

Most (87%) of the dentists had knowledge regarding the universal precautions as per OSHA regulations to be taken for treating the patients with HIV/AIDS. A large majority (82%) of the faculty members were of the opinion that HBV is more infectious than HIV and 82.0% of the dentists had opinion that infection control practices for HBV are adequate for protection against HIV. A large proportion of the faculty members (84%) were of the opinion that exposure to saliva is a highly infectious source for transmission of HIV infection to the treating dentist (Table 2).

Most (88.0%) of the dentists were aware of the immediate measures to be taken in case of a needle stick injury by taking the first-aid in the form of washing the wound with soap and water. Majority of the faculty members (60%) reported that the risk of contracting HIV infection from an HIV-contaminated needle stick injury is less than 1%. The knowledge regarding the drugs to be taken under the antiretroviral therapy (ART) was found to be good (72.0%) among the faculty members (Table 2).

The faculty members had adequate knowledge regarding the oral manifestations associated with HIV/AIDS such as oral candidiasis (95.0%), oral hairy leukoplakia (85.0%), necrotizing ulcerative gingivitis (88.0%), herpes zoster (81.0%), Kaposi's sarcoma (86.0%), Aphthous ulcers (81.0%), and salivary gland disease (75.0%). As such, there were no significant differences between the male and female faculty members with respect to the knowledge regarding oral manifestations associated with HIV/AIDS (Table 3).

## 4. Discussion

A lot of studies have been conducted regarding the knowledge and attitude towards treatment of HIV-infected individuals among the dental students and dental practitioners. However, this study evaluated the knowledge and attitude of the faculty members (dental educators) working in the dental institutions regarding the treatment of patients with HIV/AIDS. So, a direct comparison might not be possible but a comparison with dental students and dental practitioners might reveal a gap in the transmission of knowledge.

As the number of people with HIV/AIDS increases, the need of these individuals for medical care including dental care will increase [20, 21], so dental practitioners will be required to enhance their knowledge of the disease and its oral manifestations [22]. HIV-related oral conditions occur in a large proportion of individuals who are HIV-positive and frequently are misdiagnosed or inadequately treated [23]. The provision of dental care for people who are HIV-positive is essential for their overall health and well-being. Previous studies have suggested that knowledge may affect attitudes towards treatment of HIV/AIDS patients [24, 25].

The overall willingness to treat HIV-positive patients was found to be high (86%) among the faculty members in the present study. The lower willingness was reported among the dental students and dental practitioners in the studies conducted by Azodo et al. (58.8% among Nigerian dental students) [26], Hu et al. (49% recorded among Taiwanese dental students) [27], El-Maaytah et al. [15] (15% among the Jordanian dentists), 63.3% reported by Oboror et al. [28] among the Nigerian preclinical students, 63.6% among Nigerian dentists by Uti et al. [29], 78.4% among Nigerian dentists by Utomi et al. [30], Solomon et al. [31] (62% of United states (U.S.) dental school seniors), Bennett et al. [32] (67% among dentists in US), Nuttall and Gilbert [33] (84.3% among final-year dental students in the United Kingdom) and Seacat and Inglehart [34] (83% among US dental students).

The willingness among the faculty members was found to be better among the faculty members in the present study when compared to the dental students and dental practitioners. Willingness to treat is thought to be the most significant predictor of actual treatment of an HIV-positive patient [35]. Consequently, if HIV-positive individuals feel abandoned by caregivers, they are less likely to understand the need for prevention and to be motivated to protect others [36]. It is important when using the social intervention approaches to HIV prevention to avoid discrimination against people who are HIV-positive [37, 38]. The fear of treating HIV-infected patients may be due to inadequate knowledge of HIV transmission. Evidence indicates a low occupational risk for HIV infection among health care professionals [32].

A smaller proportion (32.0%) of the respondents agreed that treating an HIV positive patient places them at an increased risk of HIV transmission. This was similar to the study conducted by Crossley [38] (34% among dental practitioners in South Cheshire, United Kingdom) and Bennett et al. [32] (31% among dentists in US) but was comparatively lesser than the study conducted by McCarthy et al. [39] (63% among a national sample of dentists in Canada).

A positive attitude was displayed by the faculty members by willing to take treatment from a dentist who is treating an HIV-positive patient (72.0%). There exists longstanding limited access to healthcare for PLWHA, and negative attitude and unwillingness of the healthcare providers to treat have been cited as major barriers. Fear of HIV contagion has been found to be the primary reason behind the negative attitude and unwillingness of dental students and dentists to treat HIV/AIDS patients [40].

The fear of respondents related to dealing with staff fears, was reported with 64% of the respondents expressing concern. This was similar to the findings reported by Crossley [38]who reported almost same level of concern (59% among the respondents) and McCarthy et al. [39] (67% among dentists in Canada). In the present study, 20.0% of the faculty members reported that Infection control procedures necessary for treatment of the patients with HIV/AIDS could be time consuming and may affect the work efficiency of the practitioner whereas in the study by Crossley [38] (32%) and McCarthy et al. [39], 45% of the general dental practitioners reported that Infection control procedures required for treating patients with HIV/AIDS is a financial burden for their practice.

Majority (88.0%) of the faculty members agreed that routine dental care should be a part of the treatment of patients with HIV/AIDS. Dentists have a responsibility to provide care to HIV-infected patients, particularly because oral lesions are common among these patients [1]. Despite the importance of oral health care for people living with HIV/AIDS, many of these individuals fail to receive adequate oral health care treatment. This is particularly important in developing countries like India where patients with HIV/AIDS do not receive adequate professional oral care due to financial barriers as many such patients live at or below the poverty level [40].

There was a positive attitude among the faculty members as the majority (89.0%) of the respondents agreed that treatment of all patients should be done as potentially infectious of HBV or HIV. Not all patients know they are HIV-positive, and for those who do know, a high percentage (30%) do not inform their dentist [41, 42]. Procedures to avoid transmission between patients and dental care workers and from one patient to another should be routinely applied, regardless of whether patients are known to be HIV-positive. Dentists should be taught that universal precautions should be used with all patients, since dentists and patients themselves will not always be aware of who is HIV-positive [43].

Majority of the faculty members (87%) had proper knowledge about the universal precautions (OSHA regulations) which was similar to the study by Azodo et al. [26], (86.2% among final-year dental students in Nigeria). It is essential that every effort be made to protect both health care workers and patients from HIV exposure in the dental practice as the usual route of transmission is through an individual's contact with infected blood or other bodily fluids [6].

According to CDC (Centers for Disease Control and Prevention), Hepatitis B is 50–100 times more infectious than HIV. In the present study, 82.0% of the faculty members also agreed to the same fact. Similar findings were reported by Singh et al. [44], among dental students from Bhopal city, in which, 32.2% respondents replied that transmission of Hepatitis B can occur via saliva in comparison to only 10.6% for AIDS. A almost similar response rate was obtained in the study by Seacat and Inglehart [34] (75.9% among dental students in US) whereas a better response rate was reported by Sadeghi and Hakimi (91.6%) among dental students in Iran [45]. A better level of knowledge was present among the dental faculty members in comparison to the students especially from India whereas students from Iran demonstrated even a better knowledge than the faculty members which might be related to the dental curriculum followed by them.

A large proportion of respondents (82.0%) agreed that infection control practices for HBV are adequate for protection against HIV and other blood-borne pathogens. Similar results were obtained in the studies by Seacat and Inglehart [34] (73.7% among dental students in US) and Sadeghi and Hakimi (75.5% among Iranian dental students) [45].

Exposure to Saliva can readily transmit HIV infection was agreed upon by majority (84%) of the respondents which was similar to the study conducted by Seacat and Inglehart [34] (84%) and Samaranayake et al. [10] (100% among hospital dentists in Glasgow, UK). However, a low risk of transmission was reported in the studies by Sadeghi and Hakimi [45], in which 24.5% of Iranian dental students agreed that saliva can be a vehicle for the transmission of AIDS, Sheikh et al. [46] (33.6% undergraduate dental students in the oxford dental college, hospital and research centre, Bangalore, India) and Jian et al. [47] (36.9% among dental students in Udaipur, India). The currently available data suggests that while there may be virus in human saliva, the risk of HIV transmission is low, although blood contamination of the saliva may increase this risk [48]. The risk of transmission of HIV in the dental care setting has been reported to be low [49, 50]; however, this does not indicate a zero risk as dentists can be accidentally exposed to the virus and other blood-borne pathogens in the course of treating patients.

Most (88.0%) of the dentists were aware of the immediate measures to be taken in case of a needle stick injury by taking the first-aid in the form of washing the wound with soap and water. “Empiric” HIV postexposure prophylaxis should only be used if the risk of HIV in the source patient is high and the results of the source patient's HIV test will not be available promptly. Testing for HIV can be done in a few hours if the laboratory is prepared to do emergency testing [51].

Majority of the faculty members (60%) reported correctly that the risk of contracting HIV infection from an HIV-contaminated needle stick injury is less than 1%, whereas, in the study by Singh et al. [44], only 37.1% of the dental students reported that odds of HIV transmission after a single contaminated needle stick injury was 0.1–0.4%. There are many misconceptions about the risk of transmission through infected needles that need to be corrected. The risk of HIV transmission through accidental needle stick injury does exist although quite lower in comparison to other routes for transmission of HIV [52].

In the current study, the knowledge regarding Oral Manifestations associated with the HIV/AIDS was adequate such as Oral candidiasis (95.0%), Oral Hairy leukoplakia (85.0%), Necrotizing ulcerative gingivitis (88.0%), Herpes Zoster (81.0%), Kaposi's sarcoma (86.0%), Apthous ulcers (81.0%) and Salivary Gland disease (75.0%) which was similar to the study conducted by Sadeghi and Hakimi among Iranian dental students [45], in which, 98.1% respondents correctly identified oral candidiasis, 95.8% identified Major Aphthous, and 93.8% identified Kaposi's sarcoma. This was also similar to the study conducted by Crossley [38] among dentists working within the South Cheshire region, UK in which, 96% correctly identified Kaposi's sarcoma, 87% identified Oral candidiasis, 70% identified Acute ulcerative gingivitis, 73% identified Hairy leukoplakia and 67% identified Herpetic infections and Fotedar et al. [18], more than 73 percent of the dental students in the H. P. Government Dental College and Hospital, Shimla, India knew about the lesions strongly associated with HIV/AIDS such as Kaposi sarcoma, hairy leukoplakia, and oral candidiasis.

The knowledge regarding the oral manifestations associated with HIV/AIDS are found to be good among the dental faculty members in the present study and also similar results have been reported in the literature among the dental students and dental practitioners as well.

Many researchers therefore believe that the teaching methodology should go beyond a didactic communication and focus on problem based learning that includes small learning groups or affective component [53]. Also, incorporating psychological aspects of treating HIV/AIDS patients [54] and continuing dental education programmes should be conducted on a regular basis for updating of the knowledge of the dentists working in private practice and institutional based practice regarding the treatment of this special population.

Recent advances in HIV medical management have transformed HIV infection from a fatal and terminal disease into a chronic illness requiring continued management and careful monitoring. Consequently, there are more chances for dental health care workers to meet PLWHA in clinics, and the workers should be prepared with knowledge and the appropriate attitude [55].

However, the results of the present study cannot be generalized as it has been done on a limited sample size from two dental colleges. Moreover, the questions employed were sufficiently simple and unambiguous to achieve a reasonable degree of validity on the different variables.

## 5. Conclusion

The present study shows that there was adequate knowledge among the faculty members regarding the oral manifestations and dental treatment of patients with HIV/AIDS. The attitude of the dental faculty members was also found to be satisfactory with most of the faculty members willing to treat HIV/AIDS and considered it to be their ethical responsibility as well.

The faculty members in the dental institutions teach the dental students who might go on to become the dental faculty members in the future forming a vicious circle. So, this mandates an appropriate knowledge of the management of patients with HIV infections and should be regarded as the issues of importance in the dental education curricula. Also, a constant update is required to update the knowledge of the faculty members by attending CDE programmes and conferences related to the dental management of HIV/AIDS patients.

## Figures and Tables

**Table 1:** Showing attitude of faculty members towards the dental treatment of patients with HIV/AIDS.

	Gender		Total	*P* value
	Male	Female		
Question 1: Do you willingly treat a patient if your patient is HIV positive?				
Agree	47 (85.5%)	39 (86.7%)	86 (86.0%)	0.433
Disagree	4 (7.3%)	5 (11.1%)	9 (9.0%)	
Don't know	4 (7.3%)	1 (2.2%)	5 (5.0%)	
Total	**55** (**100.0**%)	**45** (**100.0**%)	**100** (**100.0**%)	
Question 2: Treating an HIV positive patient is ethical responsibility of the dentist?				
Agree	47 (85.5%)	32 (71.1%)	79 (79.0%)	0.190
Disagree	4 (7.3%)	8 (17.8%)	12 (12.0%)	
Don't know	4 (7.3%)	5 (11.1%)	9 (9.0%)	
Total	**55** (**100.0**%)	**45** (**100.0**%)	**100** (**100.0**%)	
Question 3: Treating an HIV positive patient places a dentist at an increased risk of HIV infection?				
Agree	23 (41.8%)	9 (20.0%)	32 (32.0%)	0.021^*^
Disagree	30 (54.5%)	36 (80.0%)	66 (66.0%)	
Don't know	2 (3.6%)	0 (0%)	2 (2.0%)	
Total	**55** (**100.0**%)	**45** (**100.0**%)	**100** (**100.0**%)	
Question 4: Will you take treatment from a doctor if you know that he/she is treating an HIV positive individual?				
Agree	41 (74.5%)	31 (68.9%)	72 (72.0%)	0.346
Disagree	9 (16.4%)	12 (26.7%)	21 (21.0%)	
Don't know	5 (9.1%)	2 (4.4%)	7 (7.0%)	
Total	**55** (**100.0**%)	**45** (**100.0**%)	**100** (**100.0**%)	
Question 5: The fear among staff/assistant towards patients with HIV/AIDS can be hindrance in providing dental care to HIV positive patients?				
Agree	33 (60.0%)	31 (68.9%)	64 (64.0%)	0.438
Disagree	16 (29.1%)	12 (26.7%)	28 (28.0%)	
Don't know	6 (10.9%)	2 (4.4%)	8 (8.0%)	
Total	**55** (**100.0**%)	**45** (**100.0**%)	**100** (**100.0**%)	
Question 6: Infection control procedures necessary for treatment of the patients with HIV/AIDS are time-consuming and may affect the work quality of the dentist?				
Agree	10 (18.2%)	10 (22.2%)	20 (20.0%)	0.042^*^
Disagree	43 (78.2%)	27 (60.0%)	70 (70.0%)	
Don't know	2 (3.6%)	8 (17.8%)	10 (10.0%)	
Total	**55** (**100.0**%)	**45** (**100.0**%)	**100** (**100.0**%)	
Question 7: Do you think that routine dental care should be a part of the treatment of patient with HIV/AIDS?				
Agree	48 (87.3%)	40 (88.9%)	88 (88.0%)	0.693
Disagree	4 (7.3%)	4 (8.9%)	8 (8.0%)	
Don't know	3 (5.5%)	1 (2.2%)	4 (4.0%)	
Total	**55** (**100.0**%)	**45** (**100.0**%)	**100** (**100.0**%)	
Question 8: Treatment of all the patients should be done as potentially infectious of HBV or HIV?				
Agree	45 (81.8%)	44 (97.8%)	89 (89.0%)	0.034^*^
Disagree	5 (9.1%)	1 (2.2%)	6 (6.0%)	
Don't know	5 (9.1%)	0 (0%)	5 (5.0%)	
Total	**55** (**100.0**%)	**45** (**100.0**%)	**100** (**100.0**%)	

^*^The *P* value is significant when less than 0.05.

**Table 2:** Showing knowledge of faculty members towards the dental treatment of patients with HIV/AIDS.

	Gender		Total	*P* value
	Male	Female		
Question 9: The universal precautions (OSHA regulations) include the use of mouth mask, gloves, protective apron and eye shields?				
Yes	47 (85.5%)	40 (88.9%)	87 (87.0%)	0.861
No	6 (10.9%)	4 (8.9%)	10 (10.0%)	
Don't know	2 (3.6%)	1 (2.2%)	3 (3.0%)	
Total	**55** (**100.0**%)	**45** (**100.0**%)	**100** (**100.0**%)	
Question 10: In your opinion, HBV is more infectious than HIV?				
Yes	45 (81.8%)	37 (82.2%)	82 (82.0%)	0.913
No	4 (7.3%)	4 (8.9%)	8 (8.0%)	
Don't know	6 (10.9%)	4 (8.9%)	10 (10.0%)	
Total	**55** (**100.0**%)	**45** (**100.0**%)	**100** (**100.0**%)	
Question 11: Infection control practices for HBV are adequate for protection against HIV and other blood borne pathogens?				
Yes	46 (83.6%)	36 (80.0%)	82 (82.0%)	0.287
No	9 (16.4%)	7 (15.6%)	16 (16.0%)	
Don't know	0 (0%)	2 (4.4%)	2 (2.0%)	
Total	**55** (**100.0**%)	**45** (**100.0**%)	**100** (**100.0**%)	
Question 12: Exposure to blood/saliva during a dental procedure can potentially transmit HIV to the treating dentist?				
Yes	41 (74.5%)	43 (95.6%)	84 (84.0%)	0.016^*^
No	12 (21.8%)	2 (4.4%)	14 (14.0%)	
Don't know	2 (3.6%)	0 (0%)	2 (2.0%)	
Total	**55** (**100.0**%)	**45** (**100.0**%)	**100** (**100.0**%)	
Question 13: Immediate measure to be taken after a needle stick injury includes?				
Wash with soap and water	49 (89.1%)	39 (86.7%)	88 (88.0%)	0.278
Take postexposure prophylaxis	4 (7.3%)	6 (13.3%)	10 (10.0%)	
Don't know	2 (3.6%)	0 (0%)	2 (2.0%)	
Total	**55** (**100.0**%)	**45** (**100.0**%)	**100** (**100.0**%)	
Question 14: What is the risk of contracting HIV infection from an HIV-contaminated needle stick injury?				
Less than 1%	33 (60.0%)	27 (60.0%)	60 (60.0%)	0.113
1–10%	5 (9.1%)	4 (8.9%)	9 (9.0%)	
11–50%	4 (7.3%)	10 (22.2%)	14 (14.0%)	
More than 50%	4 (7.3%)	2 (4.4%)	6 (6.0%)	
Don't know	9 (16.4%)	2 (4.4%)	11 (11.0%)	
Total	**55** (**100.0**%)	**45** (**100.0**%)	**100** (**100.0**%)	
Question 15: A combination of transcriptase inhibitors (NNRTIs and NRTIs) and protease inhibitors (PIs) is taken under standard antiretroviral therapy (ART) for treatment of HIV/AIDS?				
Yes	41 (74.5%)	31 (68.9%)	72 (72.0%)	0.577
No	5 (9.1%)	3 (6.7%)	8 (8.0%)	
Don't know	9 (16.4%)	11 (24.4%)	20 (20.0%)	
Total	**55** (**100.0**%)	**45** (**100.0**%)	**100** (**100.0**%)	

^*^The *P* value is significant when less than 0.05.

**Table 3:** Showing oral manifestations associated with the HIV/AIDS.

Question 16: Oral manifestations associate with the HIV/AIDS?	Gender		Total	*P* value
	Male	Female		
Question 16 a: Oral candidiasis				
Sure	52 (94.5%)	43 (95.6%)	95 (95.0%)	0.332
Not sure	1 (1.8%)	2 (4.4%)	2 (2.0%)	
Don't know	2 (3.6%)	0 (0%)	3 (3.0%)	
Total	**55** (**100.0**%)	**45** (**100.0**%)	**100** (**100.0**%)	
Question 16 b: Oral hairy leukoplakia				
Sure	48 (87.3%)	36 (82.2%)	85 (85.0%)	0.348
Not sure	3 (5.5%)	6 (13.3%)	9 (9.0%)	
Don't know	4 (7.3%)	2 (4.4%)	6 (6.0%)	
Total	**55** (**100.0**%)	**45** (**100.0**%)	**100** (**100.0**%)	
Question 16 c: Necrotizing ulcerative gingivitis				
Sure	49 (89.1%)	39 (80.0%)	88 (88.0%)	0.933
Not sure	2 (3.6%)	2 (4.4%)	4 (4%)	
Don't know	4 (7.3%)	4 (8.9%)	8 (8%)	
Total	**55** (**100.0**%)	**45** (**100.0**%)	**100** (**100.0**%)	
Question 16 d: Herpes zoster				
Sure	46 (83.6%)	35 (77.8%)	81 (81%)	0.737
Not sure	5 (9.1%)	5 (11.1%)	10 (10%)	
Don't know	4 (7.3%)	5 (11.1%)	9 (9%)	
Total	**55** (**100.0**%)	**45** (**100.0**%)	**100** (**100.0**%)	
Question 16 e: Kaposi sarcoma				
Sure	47 (85.5%)	39 (86.7%)	86 (86.0%)	0.918
Not sure	6 (10.9%)	5 (11.1%)	11 (11.0%)	
Don't know	2 (3.6%)	1 (2.2%)	3 (3.0%)	
Total	**55** (**100.0**%)	**45** (**100.0**%)	**100** (**100.0**%)	
Question 16 f: Aphthous ulcers				
Sure	44 (80.0%)	37 (82.2%)	81 (81.0%)	0.943
Not sure	5 (9.1%)	4 (8.9%)	6 (6.0%)	
Don't know	6 (10.9%)	4 (8.9%)	6 (6.0%)	
Total	**55** (**100.0**%)	**45** (**100.0**%)	**100** (**100.0**%)	
Question 16 g: Salivary gland disease				
Sure	40 (72.7%)	35 (77.8%)	75 (75.0%)	0.914
Not sure	9 (16.4%)	5 (11.1%)	14 (14.0%)	
Don't know	6 (10.9%)	5 (11.1%)	11 (11.0%)	
Total	**55** (**100.0**%)	**45** (**100.0**%)	**100** (**100.0**%)	

^*^The *P* value is significant when less than 0.05.
